# P11 Pharmacists’ knowledge, attitudes and perceptions towards antimicrobial stewardship and resistance during the COVID-19 pandemic at secondary care settings in the UK

**DOI:** 10.1093/jacamr/dlae136.015

**Published:** 2024-08-23

**Authors:** Rasha Abdelsalam Elshenawy, Nkiruka Umaru, Zoe Aslanpour

**Affiliations:** Department of Clinical Pharmacy and Pharmaceutical Sciences, School of Life and Medical Sciences, University of Hertfordshire, Hatfield AL10 9AB, UK; Department of Clinical Pharmacy and Pharmaceutical Sciences, School of Life and Medical Sciences, University of Hertfordshire, Hatfield AL10 9AB, UK; Department of Clinical Pharmacy and Pharmaceutical Sciences, School of Life and Medical Sciences, University of Hertfordshire, Hatfield AL10 9AB, UK

## Abstract

**Background:**

Addressing antimicrobial resistance (AMR) is pivotal during the COVID-19 pandemic. Inappropriate antibiotic prescribing exacerbated AMR, prompting the WHO to advocate for antimicrobial stewardship (AMS). The Royal Pharmaceutical Society (RPS) highlighted pharmacists’ role in optimizing antimicrobial use. Investigating pharmacists’ knowledge, attitudes and perceptions (KAP) regarding antibiotic prescribing, AMR and AMS contributes to global efforts to combat AMR and improve AMS practices.

**Objectives:**

To explore pharmacists’ KAP during COVID-19.

**Methods:**

In 2023, the study utilized a prospective cross-sectional design and employed an online survey questionnaire via Qualtrics to evaluate pharmacists’ KAP regarding antibiotic prescribing, AMR and AMS. Ethical approval was obtained, and implicit consent was assumed. The participants were registered pharmacists working at a secondary care setting, an NHS Foundation Trust within the UK, during the COVID-19 pandemic. The study involved patient and public involvement, was registered with the ISRCTN and OCTOPUS registry, and analysed data using descriptive statistics and IBM SPSS Statistics. A sample size of 125 was considered for a 5% margin of error and 95% confidence level.

**Results:**

The results revealed that the majority of respondents were aged 25–31 (44.0%), held postgraduate degrees (70.4%), were female (71.2%) and had 6–20 years of experience (41.6%). Regarding knowledge, high percentages agreed that antimicrobial resistance is a public health concern (85.2%), actions in combating AMR affect society (91.2%) and implementing AMS promotes judicious antibiotic use (85.6%). For attitudes and perceptions, respondent pharmacists indicated agreement with updated antibiotic guidelines (56.8%), influenced antibiotic prescribing due to COVID-19 patient conditions (80%) and support from microbiologists and AMS teams for informed decisions (79.2%).

**Conclusions:**

This study highlights pharmacists’ crucial role in fighting AMR during the COVID-19 pandemic. They demonstrated good KAP emphasizes their potential to co-lead AMS implementation, ultimately saving lives and promoting global health.
